# Association of High-Speed Rail and Tuberculosis Transmission in Newly Integrated Regions: Quasi-Experimental Evidence from China

**DOI:** 10.3389/ijph.2021.1604090

**Published:** 2021-11-11

**Authors:** Yahong Liu, Chengxiang Tang, Tao Bu, Daisheng Tang

**Affiliations:** ^1^ School of Economics and Management, Beijing Jiaotong University, Beijing, China; ^2^ School of Public Administration, Guangzhou University, Guangzhou, China

**Keywords:** public health, China, high-speed railway, tuberculosis, public transportation, the tiered-network healthcare policy

## Abstract

**Objectives:** The spread of tuberculosis (TB) is related to changes in the social network among the population and people’s social interactions. High-speed railway (HSR) fundamentally changed the integrated market across cities in China. This paper aims to examine the impact of HSR on TB transmission in newly integrated areas.

**Methods:** By exploiting the opening and operation of the first HSR in Sichuan province as a quasi-natural experiment, we have collected and used the economic, social, and demographic data of 183 counties in Sichuan province from 2013 to 2016.

**Results:** The new HSR line is associated with a 4.790 increase in newly diagnosed smear-positive TB cases per 100,000 people among newly integrated areas. On average, an additional increase of 34.178 newly diagnosed smear-positive TB cases occur every year in counties (or districts) covered by the new HSR.

**Conclusion:** HSR development has significantly contributed to the transmission of TB. The public health system in China needs to pay more attention to the influences of new, mass public transportation.

## Introduction

As the third largest country with a high risk burden of tuberculosis (TB) in the world following India and Indonesia, TB is one of the major public health problems in China. According to the Global TB Report, China reports about 900,000 new cases, 40,000 deaths, and 70,000 cases of drug-resistant TB every year [1]. From 2012 to 2018, the incidence of TB per 100,000 capita in China dropped from 70.6 to 59.3, and the treatment success rate remained above 90 percent. However, the current epidemic situation of TB in China is still serious, with the number of new cases reported each year and the incidence rate per 100,000 capita ranking second among category A and category B infectious diseases, after viral hepatitis, and well above other category A and category B infectious diseases (such as syphilis and gonorrhea) [2]. TB is an infectious disease caused by the mycobacterium TB (MTB), which is transmitted through the air by coughing, sneezing, and spitting by patients with TB [3]. As one of the infectious agents of TB, sputum smear-positive TB patients are highly infectious and more likely to develop drug-resistant/multidrug-resistant TB than sputum smear-negative patients. The spreading of TB is related to the number and nature of the contact between infectious and healthy people, social networks among the population, and how social interactions change [4–6].

In China, the incidence and transmission of TB is related to age, occupation, and genetic susceptibility at the individual level, and related to socio-economic factors, the natural environment, and input and distribution of medical and health resources at the population level. Ethnic minorities, the agricultural population, and residents in economically underdeveloped areas are at a higher risk of TB infection due to the lack of education and nutrition, inadequate health protection, and fewer medical resources. Especially, TB control of the floating population is a major problem. In 2020, a floating population of 376 million, out of a total population of 1.41 billion (The Seventh National Census, 2020), moved from region to region in search of better income and living conditions. They are a high-risk group of TB compared to the local population due to low income and education, poor health awareness, low social insurance or medical insurance coverage, and poor access to medical services and medical resources [7]. Patients in the floating population are more likely to delay medical treatment or give up treatment [8]. Non-standard treatment often occurs in the cross-regional referral of confirmed floating population pulmonary TB patients, and even the treatment is interrupted. In addition, screening of close TB contacts is an important means of proactively detecting TB patients, but it has not been effective in China.

Historically, major transitional periods of human travel and population mixing have been accompanied by large-scale outbreaks of disease and spread of pathogens [9, 10]. Measures to limit interpersonal, social, and economic activity, such as the closure of schools or public transport networks, rail strikes, and general curfews, were effective in controlling the spread of influenza, gastroenteritis, and chicken pox in France from 1984 to 2010. These measures to reduce personal contact resulted in a significant decline in the rate of disease transmission. However, infectious diseases spread faster in prosperous times, and improved transport networks may have a negative impact on people’s health through frequent interpersonal interactions in the region, leading to a worse epidemiological environment [5]. Densely populated and poorly ventilated environments can trigger a significant risk of infection, such as TB, as documented in various forms of public transportation elsewhere (rail, sea/water, buses, minibus taxis, subways, etc.) [11–15]. The development of roads, highways, railways, and air travel may increase the speed and volume of disease transmission [16], and the cost of these transportation infrastructures in spreading disease is a costly externality that leads to health care and productivity lost, and has a long-term impact on economic growth [4, 17–19].

The high-speed railway (HSR) provides a cheaper and more energy-efficient way for mass public transportation than vehicles. HSR is particularly suitable for high population-density areas, such as the Eurasian continent. HSR in China has developed rapidly since 2008, which mainly consists of a network of passenger-specific railways with high speeds between 250 and 350 km per hour. By the end of 2019, the national HSR network had extended to 32 out of 34 provincial-level regions, with a total length of 35,000 km, which accounts for around two-thirds of the commercial HSR tracks in the world [20]. In addition, the volume of passenger traffic has been increasing. Of the 3.57 billion passenger trips delivered by the China Railway network in 2019, the HSR train carried out 2.29 billion passenger trips. After a decade of development, the HSR has been considered an ideal approach for population mobility in China. The universal acceptance of HSR in China represents a strategical transformation from road-air mixed transportation to rail-dominant transportation, and thus integrates more and more regional markets.

During the last decade, rapid improvements in public transportation and related infrastructure have increased population movement and promoted cross-regional economic activities. At the same time, it has potential risks for public health, for example, the spread of communicable diseases [5, 21]. Consistent with economic development, both the absolute and relative health disparities in TB across provinces increased from 1990 to 2016 in China [22]. Following the outbreak of acute respiratory syndrome (SARS) in 2003, China re-established its public health system and rewrote a significant number of policies pertaining to public health. The public health system has also contributed to the quick containment of COVID-19 in the spring of 2020 [23]. However, communicable disease spreading remains a major threat to human life and health. The key to controlling these diseases is to raise public awareness and to develop more effective monitoring and preventive measures [24, 25].

The construction of HSR has fundamentally changed the transport infrastructure and integrated market across cities in China. Despite tremendous changes in both public transport mode and population mobility, there is still little evidence presented on the association between public health and development of the HSR. There is still a lack of quantification and direct assessment of the risk [26, 27]. Any event that leads to changes in population and social interaction has the potential to affect the spread of disease, but epidemiological work rarely provides direct and data-based evidence on the effectiveness of policies aimed at reducing exposure [4]. This paper aims to examine whether, and if so to what extent, the establishment of the HSR has increased the transmission of TB in the areas newly covered by the HSR.

## Methods

### Setting and Data

**FIGURE 1:**
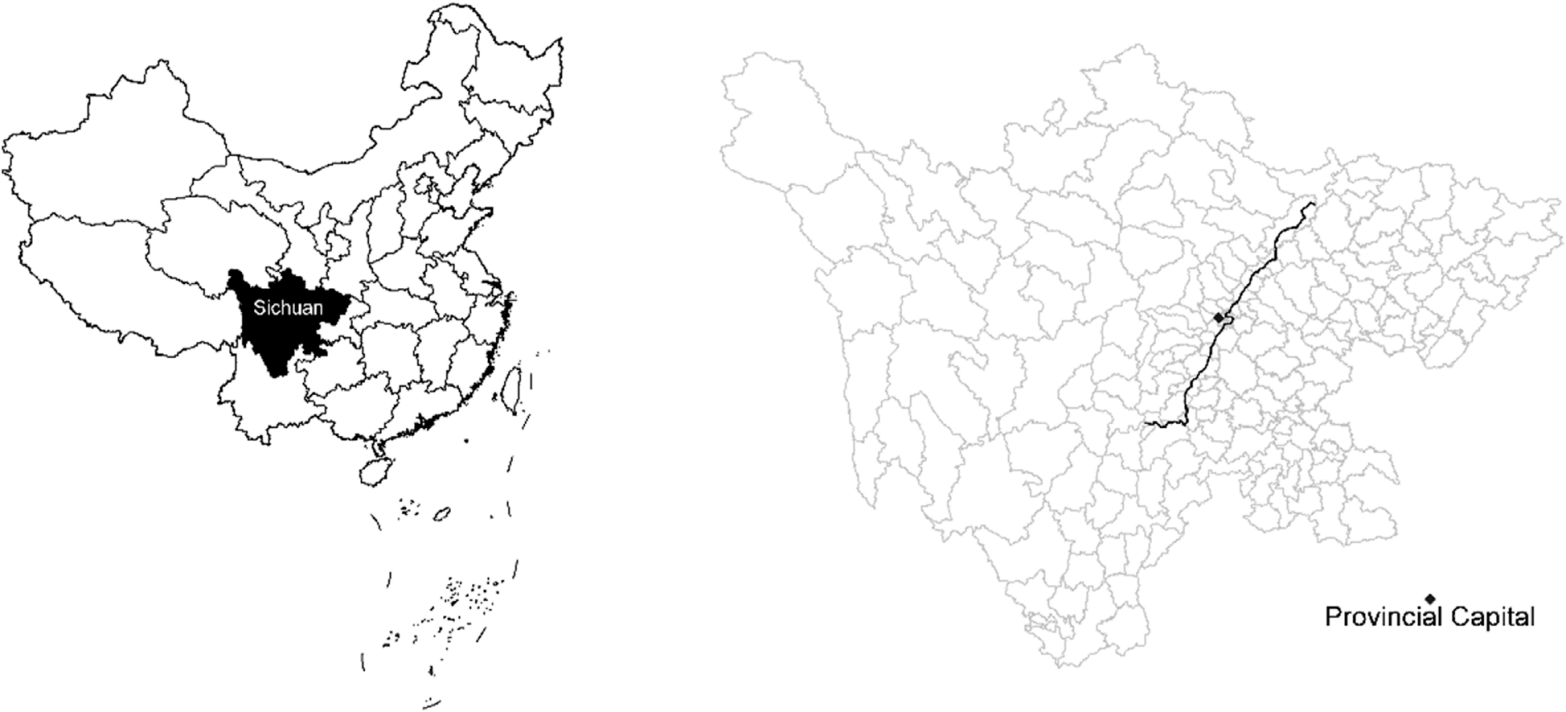
Sichuan province and Cheng-Mian-Le high-speed rail along counties (or districts), China, 2013–2016.

Figure 1 illustrates the position and areas of Sichuan province in China with populations over 86 million. One of the important reasons we chose this place is that the province can be considered a remote area compared to the eastern coastal regions. The first HSR line was not opened until December 20, 2014. The first HSR line in Sichuan province, namely the Cheng-Mian-Le HSR Line for passenger transportation, was designed to run at the speed of 250 km per hour, connecting sixteen counties (or districts) from north to south. In addition, given the geographical makeup, a basin, the province is a relatively closed-in area and thus it is an ideal environment to examine the public health impacts of the HSR. The opening and operation of the Cheng-Mian-Le HSR marked an era of a new mode of mass public transportation in Sichuan province, which provided an appropriate “quasi-natural experiment” setting of external shock.

Among the 183 counties (or districts) in Sichuan province, we removed 14 counties as they are covered by the Chengdu-Chongqing HSR that began operation on December 26, 2015, so that it will not cause confounding factors in our setting. Using the remaining 169 counties (or districts), the study has obtained a panel of data of 676 samples from 2013 to 2016. We defined the period from 2013 to 2014 as the non-experimental period, and the value of time dummy variable (dum_year) as 0; we defined the period from 2015 to 2016 as the experimental period, and the value of time dummy variable as 1. The 16 counties (or districts) covered by HSR are the treatment group, and the value of the dummy variable of HSR (dum_HSR) is 1; the 153 counties (or districts) not covered by HSR are the control group, and the value of the dummy variable of HSR is 0.

The Sichuan Statistical Yearbook of Health and Family Planning contains publicly available data on disease control and public health compiled by the Sichuan Provincial Health Commission. The number of new smear-positive TB patients and cured cases of the 183 counties (or districts) in Sichuan province has been recorded every year. We are concerned with two TB transmission indicators: the number of newly diagnosed smear-positive TB cases ( $TB_{i,t}$ ) and the incidence of newly diagnosed smear-positive TB cases per 100,000 capita ( $TB\_rate_{i,t}$ ) in the county (or district) of Sichuan from 2013 to 2016. The incidence of smear-positive TB cases per 100,000 capita is calculated by dividing the number of smear-positive TB cases by the number of permanent residents at the end of the year. All other control variables are also from the statistical yearbook.

### Modeling

The difference-in-difference model (DID) has significant advantages in dealing with endogenous issues caused by causal identification and variable omission. The impact of public transportation on regional social and economic development usually indicates the problem of mutual causal identification, because whether some areas have built HSR infrastructure is not entirely exogenous. In order to avoid sample selection bias, this paper also adopts the propensity score matching with difference-in-difference model (PSM-DID) to evaluate the net effect of the new HSR line on TB transmission. The Probit model is used to estimate the propensity score. Kernel matching is used to determine the weight.

**FIGURE 2:**
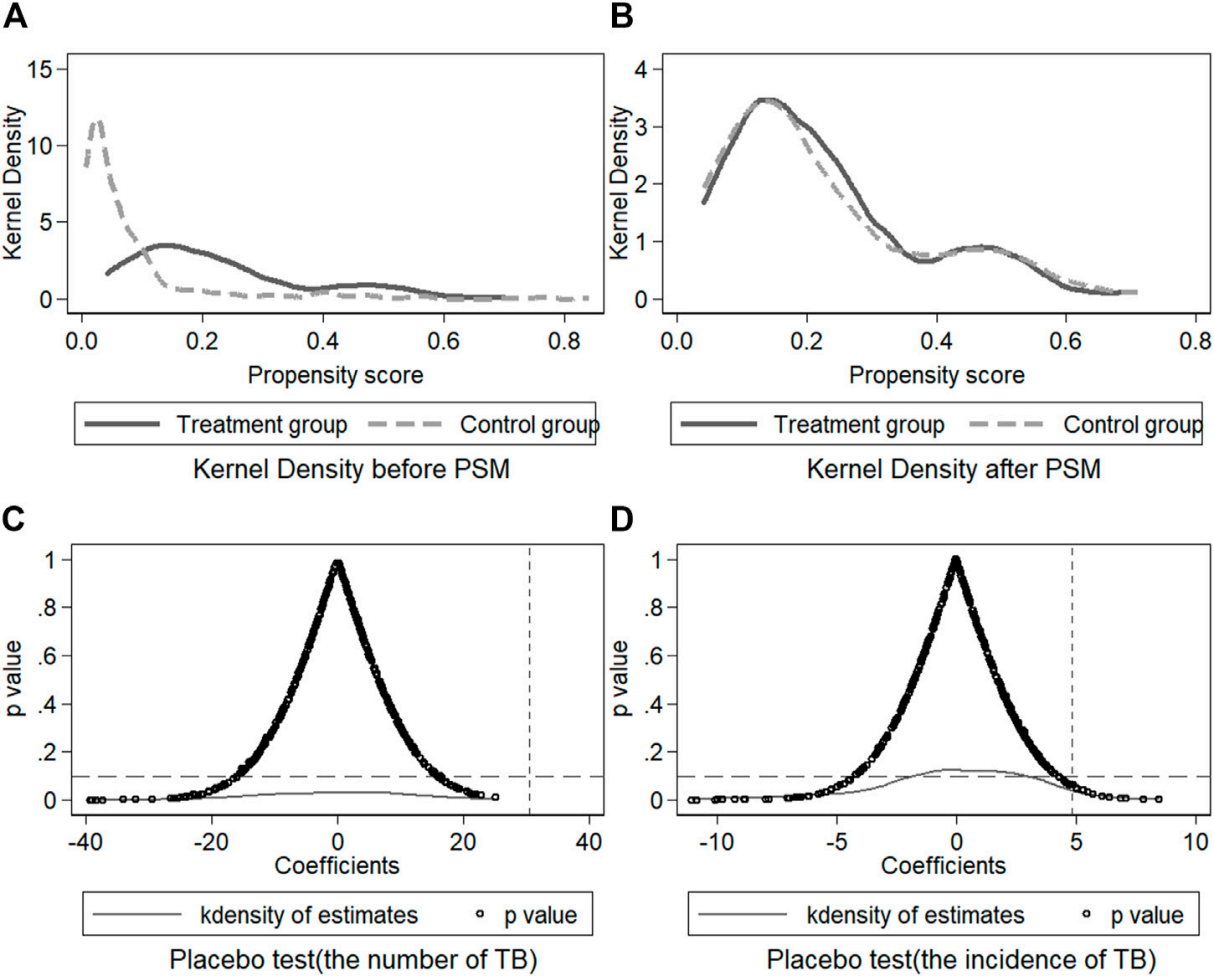
Kernel density of the treatment and control group and placebo test, Sichuan Province, China, 2013–2016.

There are five variables used to estimate the propensity scores between counties with HSR and those without, thus this study has obtained scores from the control group similar to the treatment group [28]. GDP per capita measures the economic development level, population density measures the degree of population aggregation, urbanization rate measures the level of urbanization, the proportion of employees in the tertiary industry measures the employment structure, and the ratio of GDP of the secondary industry (industrial structure) measures the structure of economic development in a county. Figures 2A,B shows that the distribution of TB incidence kernel density is consistent after matching using the PSM for both the treatment group and control group.

The baseline models are as follows:

$$TB_{i,t}=\alpha_{0}+\alpha_{1}*HSR_{i,t}+\varphi_{n}\sum_{n=1}^{n}X_{i,t}+\delta_{i}+\mu_{t}+\varepsilon_{it}$$ (1)

$$TB\_rate_{i,t}=\alpha_{0}+\alpha_{1}*HSR_{i,t}+\varphi_{n}\sum_{n=1}^{n}X_{i,t}+\delta_{i}+\mu_{t}+\varepsilon_{it}$$ (2)

$$HSR_{i,t}=dum\_HSR_{i,t}*dum\_year_{i,t}$$ (3)

The coefficient $\alpha_{1}$ is the net effect of the opening of HSR on the transmission of TB. $X_{i,t}$ is the same set of control variables as PSM. $\delta_{i}$ is the region-fixed effect, $\mu_{t}$ is the time-fixed effect, and $\varepsilon_{i,t}$ is the residual term.

In order to test whether the update of transportation mode provides a more efficient way for population mobility, we use region passenger-kilometer (PKM) to measure the frequency of social interaction-related population flow. PKM is the passenger-distance travelled by passengers on transit vehicles that is determined by multiplying the number of trips by the average length of their trips. We consider that HSR will change the aggregation degree of social and economic exchanges of the population who live along the HSR line, and thus we infer that the introduction of HSR affects TB transmission by affecting PKM in the region [27].

$$TB{\left(or\;TB_{rate}\right)}_{i,t}=\beta_{0}+\beta_{1}dum\_HSR_{i,t}*PKM_{i,t}+\beta_{2}dum\_HSR_{i,t}+\beta_{3}PKM_{i,t}+\theta_{n}\overset{n}{\underset{n=1}{\sum }}X_{i,t}+\delta_{i}+\mu_{t}+\varepsilon_{i,t}$$ (4)

$\beta_{1}$ measures the incremental impact of PKM on the transmission of TB in areas covered by HSR compared to areas without. $\beta_{1}+\beta_{3}\;$ measures the impact of changes in PKM on TB transmission in areas covered by HSR.

Descriptive statistics is available in Appendix Table 1. Variance inflation factor (VIF) can measure the severity of multicollinearity. The variance inflation factor corresponding to each variable was all lower than 10 (see Appendix Table 2), experience shows that there was no multicollinearity.

## Results

### Benchmark Regression and Robust Test

In Table 1, the dependent variable for models [1–3] is the number of newly diagnosed smear-positive TB cases (TB), the dependent variable for models [4–6] is the incidence of newly diagnosed smear-positive TB per 100,000 capita (incidence of TB). Model [3] shows that the counties (or districts) covered by HSR have 34.178 more TB cases than the counties (or districts) without, and model [6] shows that the incidence of TB in counties (or districts) with HSR line is 4.790 higher than that of the counties (or districts) without HSR on average. The results indicate that the opening and operation of HSR strengthen TB transmission in newly integrated areas.

**TABLE 1:** Impact of Cheng-Mian-Le high-speed rail on tuberculosis transmission, Sichuan province, China, 2013–2016.

	TB			Incidence of TB		
	[1] DID	[2] DID	[3] PSM-DID	[4] DID	[5] DID	[6] PSM-DID
Impacts of HSR	32.460**	37.933**	34.178**	6.188**	6.110**	4.790*
	(15.913)	(16.497)	(16.703)	(2.728)	(2.677)	(2.629)
GDP per capita		−6.879***	-0.213		0.121	0.547*
		(1.661)	(2.015)		(0.327)	(0.327)
Population density		0.009***	0.001		0.001***	−0.000
		(0.001)	(0.002)		(0.000)	(0.000)
Urbanization rate		−0.362	−0.427		−0.222***	−0.125***
		(0.242)	(0.281)		(0.045)	(0.047)
Employment structure		1.955***	0.255		−0.041	−0.139**
		(0.273)	(0.399)		(0.055)	(0.060)
Industrial structure		0.084	−1.289***		0.079	−0.034
		(0.190)	(0.378)		(0.048)	(0.054)
Cons	74.415***	61.097***	174.903***	20.201***	24.685***	27.793***
	(4.185)	(9.565)	(23.671)	(1.067)	(2.148)	(3.276)
N	676	676	370	676	676	370
R^2^	0.034	0.136	0.133	0.028	0.060	0.086
Control variables	No	Yes	Yes	No	Yes	Yes
Time-fixed	Yes	Yes	Yes	Yes	Yes	Yes
Region-fixed	Yes	Yes	Yes	Yes	Yes	Yes

The reported value refers to the coefficient of the explanatory variable, and the *p* value is measured by the superscript of the coefficient: *, **, and *** are significant at 10, 5, and 1% levels, respectively. The numbers in brackets are the robust standard errors for clustering to counties (or districts).

We further tested the robustness of baseline regression results through the placebo test, including a policy time simulation with the HSR opening date assumed to be 2013, and a HSR counties (or districts) simulation with a random sample of 400 times by the bootstrap method. The results (*p* value) of the placebo test (see Table 2A and Figures 2C,D) show that there was no significant effect on TB transmission in counties (or districts), which proved the robustness of the results.

**TABLE 2:** Robustness test and mechanism test on the impact of high-speed rail on tuberculosis transmission, Sichuan province, China, 2013–2016.

A. Robustness test (placebo test of opening time of high-speed railway)				
	TB		Incidence of TB	
	[7] DID	[8] PSM-DID	[9] DID	[10] PSM-DID
HSR	21.459	24.099	5.030	3.384
	(17.553)	(19.078)	(2.974)	(2.807)
Cons	79.810***	178.459***	21.796***	28.419***
	(6.293)	(24.622)	(1.589)	(3.385)
*N*	676	370	676	370
*R* ^2^	0.030	0.125	0.026	0.080

**B. The mediation effect of region passenger kilometers**				
	TB		Incidence of TB	
	[11] DID	[12] PSM-DID	[13] DID	[14] PSM-DID
HSR* PKM	0.091	0.053	0.062**	0.028**
	(0.083)	(0.088)	(0.024)	(0.012)
HSR	−8.581	9.091	−14.313***	−5.441***
	(14.649)	(12.925)	(5.085)	(1.983)
PKM	−0.101	−0.084	−0.046***	−0.018**
	(0.065)	(0.073)	(0.016)	(0.009)
Cons	52.063***	144.566***	23.088***	27.114***
	(7.196)	(24.780)	(2.853)	(3.949)
*N*	676	370	676	370
*R* ^2^	0.436	0.323	0.206	0.228
Control variables	No	Yes	No	Yes
Time-fixed	Yes	Yes	Yes	Yes
Region-fixed	Yes	Yes	Yes	Yes

The reported value refers to the coefficient of the explanatory variable, and the *p* value is measured by the superscript of the coefficient: *, **, and *** are significant at 10, 5, and 1% levels, respectively. The numbers in brackets are the robust standard errors for clustering to counties (or districts).

### The Mediation Effect of Region Passenger Kilometers

In Table 2B, we measure the dynamic characteristics of the floating population by PKM. $\beta_{1}$ , yielding the result 0.028, which is significantly positive, indicates that the incidence of TB in areas covered by HSR is more sensitive to changes in PKM than the areas without. $\beta_{1}+\beta_{3}\;$ is 0.01, $\beta_{1}$ and $\beta_{1}+\beta_{3}\;$ are significantly positive, indicating that HSR can increase the incidence of newly diagnosed smear-positive TB per 100,000 capita in HSR areas by changing the PKM.

### Tuberculosis Situation in China and Sichuan Province

**FIGURE 3:**
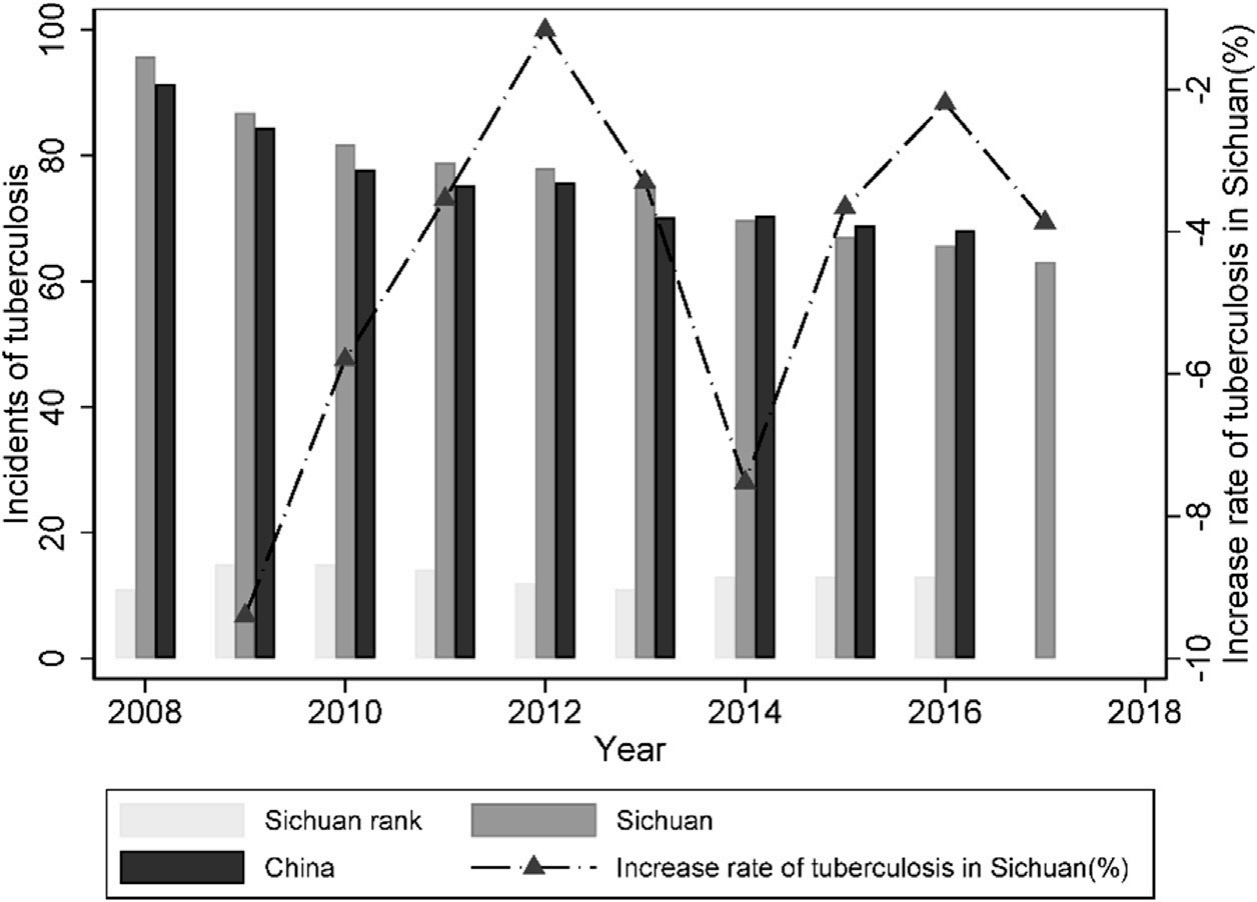
The incidence of newly diagnosed smear-positive TB per 100,000 people, Sichuan province, China, 2008–2017.

In Figure 3, the TB incidence shows a downward trend in China from 2008 to 2017, especially before 2012, which can be attributed to effective public health regulations, the long-term vaccination program, and promptness of the diagnosis and reporting system [29]. However, the trend of TB incidence has no significant change after 2012, which is also consistent with the timing of the fundamental HSR development and its related population flow. For Sichuan province, the change rate of TB incidence per 100,000 capita became much smaller from 2014 to 2016 (from −7.52% to −2.19%). This phenomenon may reflect that our hypothesis on the influences of opening and operation of HSR can be tested, because the first HSR was opened at the end of 2014 in Sichuan province.

## Discussion

### HSR Impacts on TB and Health

We have first shown a negative short-term impact of the HSR on TB transmission in China, the results are consistent with a previous study. Tang examined the large-scale expansion of Japanese railways at the end of the 19th century, and found that the average annual mortality in newly-covered areas by railways increased by 169 per 100,000 capita; 75% of mortality related to rail was attributed to infectious disease spread, including TB and influenza [28]. Even though the public health system has made great updates since the twentieth century, the introduction of a new HSR can still influence TB transmission in a direct or indirect manner.

Health improvements on the basis of transportation networks are generally from two sources. The first one is macroeconomic growth promoted by high-speed rail, which boost governments’ capability to invest in public heath, medical care and medical aid, and other medical services. The second one is to raise living standards, including access to more and better foods, and access to more human capital investment. The investment in transport infrastructure has beneficial effects such as easing congestion and boosting economic growth, while increased productivity may result in health costs associated with the spread of infectious diseases [5, 30]. Temporal and spatial changes inherent in the transition from a traditional to an industrialized economy can also affect mortality, especially if labor mobility increases the frequency of infectious diseases [28, 31].

In terms of the mechanism of TB transmission, we found that the opening of HSR has increased TB transmission in areas covered by HSR by increasing population mobility and social interaction networks. With the passing of time and the expansion of the railway across regions, the population can form more convenient and rapid regional flows as the HSR network is widely developed. While increased transport corridors may help ease traffic, foster related agglomeration and economies of scale, promote commercial and economic development, and achieve market integration, better transportation links and unrestricted mobility still increase the spread of infectious diseases such as TB and flu [32].

China has been experiencing increased urbanization, rapid population movement, and migration between different parts of the world. It is common for people to move for a better job or lifestyle [33]. With the development, more and more people travel, which increases interpersonal communication and the transmission of infectious disease [5, 34]. Population movement creates new environments and challenges for the control of infectious diseases. As immigrants and long-stay travelers or workers become a larger part of the social population, the impact of potential infections on public health could become an important issue in the region, with a focus on prevention or early detection [35, 36]. At present, China still has a number of patients with poor access to new and effective diagnoses and treatments, and with limited financial protection [37, 38]. Routine public health surveillance should include information related to the movement and migratory history of subjects that will help health care workers to understand and manage the spread of disease.

### Spatial Correlation of Tuberculosis

The occurrence and prevalence of infectious diseases are closely related to geographical and spatial information [8]. We estimated the spatial epidemiological characteristics of TB transmission in Sichuan province based on the spatial difference-in-difference model (SDID) [39]:

$$TB{\left(or\;TB_{\mathrm{rate}}\right)}_{i,t}=\rho_{0}+\rho_{1}W*TB_{i,t-1}+\rho_{2}*HSR_{i,t}+\gamma_{1}\sum_{n=1}^{n}X_{i.t}+\gamma_{2}W*\sum_{n=1}^{n}X_{i.t-1}+\delta_{i}+\mu_{t}+\varepsilon_{it}$$ (5)

Considering the degree of economic closeness between regions, the average annual GDP of each region is taken as the measuring index of economic distance between regions based on the weight matrix of geographical distance, so the weight matrix W of economic space is calculated. Spatial auto-correlation is judged by global Moran’s I. Moran’s I (see Appendix Table 3) of the number of TB cases from 2013 to 2016 ranged between 0.315 and 0.379 (*p* < 0.01), there is spatial agglomeration, that is, regional agglomeration with a high (or low) number of TB cases in space. There was no significant spatial auto-correlation of incidence per 100,000 capita.

Firstly, it is concluded that the spatial Durbin model (SDM) is superior to spatial autoregressive (SAR) and the spatial errors model (SEM) through the spatdiag deterministic LM test. Secondly, the fixed effect model is better than the random effect model through the Hausman test. Thirdly, it is concluded that SDM cannot degenerate into SAR and SEM through the LR test. Therefore, the spatial difference-in-difference Durbin model of the individual and time bidirectional fixed effect is used to estimate the spatial correlation of TB transmission between regions.

The results (see Table 3A) showed that compared with the baseline regression results, the impact of the opening of HSR on the number of TB patients and the incidence rate per 100,000 capita did not change significantly, which proves the robustness of the results. The coefficient of the spatial lag term of the number of TB cases in counties (or districts) was significantly positive, indicating that the number of TB cases in a certain county (or district) in Sichuan was higher, and the number of TB cases in other counties (or districts) with close economic ties was also higher. However, there was no spatial agglomeration in the incidence of TB per 100,000 population. The coefficient of the spatial lag term of the dummy variable of HSR opening was not significant, indicating that the opening of HSR in one county (or district) had no significant impact on the transmission of TB in other counties (or districts). This also indicates that compared with the counties (or districts) without HSR, the TB transmission in the counties (or districts) with HSR was significantly increased, mainly due to the increased population mobility and aggregation caused by the operation of HSR in the region, which leads to a higher risk of TB transmission.

**TABLE 3:** Estimation of spatial correlation of tuberculosis and the impact of the tiered-network healthcare policy on tuberculosis transmission, Sichuan province, China, 2013–2016.

A. Spatial correlation of tuberculosis				
	TB		Incidence of TB	
	[15] SDID	[16] SDID	[17] SDID	[18] SDID
HSR	36.679***	35.024***	5.993***	5.700**
	(8.523)	(8.600)	(2.282)	(2.354)
W*TB (or TB_rate)	0.122*	0.103	−0.059	−0.053
	(0.064)	(0.065)	(0.062)	(0.062)
W*HSR	−23.560	10.893	0.871	3.317
	(17.081)	(19.379)	(4.559)	(5.209)
Variance	959.408***	883.765***	69.170***	66.189***
	(52.206)	(48.085)	(3.763)	(3.600)
*N*	676	676	676	676
*R* ^2^	0.005	0.119	0.002	0.005
Control variables	No	Yes	No	Yes

**B. The tiered-network healthcare policy**				
	TB		Incidence of TB	
	[19] DID	[20] PSM-DID	[21] DID	[22] PSM-DID
HSR	37.852**	34.169**	6.084**	4.788*
	(16.501)	(16.678)	(2.674)	(2.626)
The tiered-network healthcare policy	−8.784	-12.321	-2.791	−2.074*
	(7.404)	(8.885)	(1.923)	(1.254)
Cons	65.673***	182.203***	26.139***	29.022***
	(10.401)	(24.637)	(2.428)	(3.398)
N	676	370	676	370
R^2^	0.138	0.138	0.064	0.092
Control variables	Yes	Yes	Yes	Yes
Time-fixed	Yes	Yes	Yes	Yes
Region-fixed	Yes	Yes	Yes	Yes

The reported value refers to the coefficient of the explanatory variable, and the *p* value is measured by the superscript of the coefficient: *, **, and *** are significant at 10, 5, and 1% levels, respectively. The numbers in brackets are the robust standard errors for clustering to counties (or districts).

### The Tiered-Network Healthcare Policy

The most important confounding factor in this study is the tiered-network healthcare policy. Sichuan province began to implement a tiered-network healthcare policy on October 1, 2014. The policy set up reimbursement procedures and limits to patients’ medical insurance expenses, enforced primary diagnosis at the primary level in a quasi-compulsory manner, and thus affected bypassing behavior and hospital shopping of residents. In order to prevent a biased estimation of HSR net effect, this paper further takes the policy events into account.

This paper adds the dummy variable of the tiered-network medical care system into the benchmark regression model. The dummy variable value of the tiered-network medical care system before 2013 is assigned 0, and the dummy variable value of the tiered-network medical care system in 2014–2016 is assigned 1.

In Table 3B, the results of opening and operation of HSR significantly increases the number of TB patients and the TB incidence per 100,000 capita in counties (cities or districts) covered by HSR after implementation of the tiered-network medical care system. The estimated coefficient of implementation of the tiered-network medical care policy on TB infection is significantly negative, which indicates that implementation of the tiered-network medical care policy has reduced TB infection in China, to a certain extent. However, we also can find that the number of TB patients in the extended regression model is lower than that of the benchmark regression model in Table 1, and the TB incidence per 100,000 capita in counties (cities or districts) in the extended regression model is less than that of the benchmark regression model, which demonstrates that the tiered-network medical care system has a moderate effect but not an obvious one. This shows that the tiered-network medical care system has lag effects, meanwhile, intervention of the tiered-network healthcare policy cannot work effectively after the introduction of a HSR line.

### Limitations

This study has several limitations. First, the present paper only takes the opening and operation of Cheng-Mian-Le HSR in Sichuan province as a case study of China, which may ignore external validity to some extent. Second, this study tests the regional level impact of the opening and operation of HSR on TB infection, if micro data can be obtained in the future, we can further conduct empirical analysis based on micro data. This paper also does not take into account seasonal characteristics, geographical differences, population gender differences, economic cycles, regional trade, and other influential factors [5]. Third, due to China’s restrictions on public data in health care, we did not discuss nosocomial and community TB infection. The factors mentioned before are important for understanding the long-term relationship between epidemics and socioeconomic activities. Further research regarding the on-going effects of HSR on health outcomes and the entire health system is still required, as well as on improved quality of life or mortality.

### Conclusion

Based on the DID and PSM-DID models, we estimated the impact of HSR on TB transmission in Sichuan province from 2013 to 2016. It is the first study that identified a significant increase in the incidence of newly diagnosed smear-positive TB per 100,000 capita among newly integrated areas because of HSR development. On average, an additional increase of 34.178 newly diagnosed smear-positive TB cases occur every year in counties (or districts) covered by the new HSR. Beyond the health impacts, we found that HSR can increase the incidence of newly diagnosed smear-positive TB cases per 100,000 capita in HSR areas by changing PKM, which measured the dynamic characteristics of the floating population. Furthermore, we found that there is spatial agglomeration in the newly diagnosed smear-positive TB cases in Sichuan, and the tiered-network healthcare policy cannot work effectively after the introduction of a HSR line. In conclusion, HSR development has significantly contributed to TB transmission, because the HSR access promotes the inter-regional population flow and the economic and social activity by reducing space-time distance. Both the United Nations and the World Health Organization have released a sustainable development goal and strategy to eliminate TB by 2030. The present paper has practical implications, as it can help policymakers to pay more attention to prevention and control of TB infection and relevant communicable diseases because of developments in public transportation.

## Appendix

**Appendix Table 1:** Descriptive statistics.

Variables	All Samples			Treatment Group			Control Group		
	Obs	Mean	Std.Dev.	Obs	Mean	Std.Dev.	Obs	Mean	Std.Dev.
TB(TB)	676	67.69	64.79	64	91.94	60.88	612	65.15	64.71
Incidence of TB	676	17.4	15.78	64	13.42	9.66	612	17.82	16.23
HSR	676	0.05	0.21	64	0.5	0.5	612	0	0
GDP per capita	676	3.38	2.1	64	5.61	1.64	612	3.15	2
Population density	676	716.46	1974.12	64	2192.35	3628.01	612	562.12	1642
Urbanization rate	676	41.24	18.23	64	61.25	17.85	612	39.15	16.97
Employment structure	676	20.74	12.17	64	32.18	8.45	612	19.54	11.88
Industrial structure	676	48.71	16.32	64	53.15	13.53	612	48.24	16.53

**Appendix Table 2:** Variance inflation factor (VIF) test.

Variable	VIF	1/VIF
Urbanization rate	4.250	0.235
GDP per capita	3.520	0.284
Population density	2.770	0.361
Employment structure	2.110	0.474
Industrial structure	2.050	0.488
Mean VIF	2.94	

**Appendix Table 3:** Global spatial autocorrelation test.

	TB			Incidence of TB		
	Moran’s I	Std.	*p*-value	Moran’s I	Std.	*p*-value
2013	0.328	0.040	0.000	−0.010	0.034	0.905
2014	0.315	0.040	0.000	−0.036	0.035	0.383
2015	0.379	0.040	0.000	−0.008	0.036	0.965
2016	0.346	0.040	0.000	0.077	0.040	0.037
